# More Treatment, but Not Less Anxiety and Mood Disorders: Why? Seven Hypotheses and Their Evaluation

**DOI:** 10.1159/000528544

**Published:** 2023-02-06

**Authors:** Johan Ormel, Paul M.G. Emmelkamp

**Affiliations:** ^a^University of Groningen, University Medical Center Groningen, Groningen, The Netherlands; ^b^Netherlands Institute for Advanced Study, Amsterdam, The Netherlands; ^c^Paris Institute for Advanced Study, University of Amsterdam, Amsterdam, The Netherlands

**Keywords:** Common mental disorder, Population-level impact of treatment, Treatment gaps, Counterproductive effects

## Introduction

Mood and anxiety disorders are not only common and responsible for much functional disability [1], but epidemiological studies also indicate that their prevalence, circa 10% in Western countries, has not fallen since the 1970s despite the development of evidence-based treatments [2, 3, 4, 5, 6, 7, 8]. Prevalence refers to the percentage of adults in the general population that meet diagnostic criteria in a defined period, usually the 30 days (point prevalence) or 12 months (12-month prevalence) preceding the examination irrespective of possible earlier episodes.

In sharp contrast, multiple studies have documented substantial increases in expenditures on mental health care and in treatment rates in Western countries [9, 10, 11, 12, 13, 14, 15]. The evidence on increased treatment rates comes from both general practice [16, 17, 18], nation-wide morbidity registrations [19, 20], and repeated population-based surveys [8, 21]. The treatment rate increase was bolstered by the introduction of a new class of drugs in the 1980s, the selective serotonin reuptake inhibitors, aggressively marketed by Big Pharma [22]. In addition, a number of evidence-based psychological treatments became available for people with mood and anxiety disorders.

The trend data on prevalence and treatment rate reveal a remarkable paradox: more treatment but not less disorders, the treatment-prevalence paradox. The expectation to see a declining trend in the prevalence of mood and anxiety disorders with an increasing trend in treatment is not unfounded. Treatment seeks to shorten illness episodes, prevent worsening and the development of comorbidity, reduce relapses and curtail recurrences. If effective, increased treatment rates should result in lower prevalence rates in the general population, but this prevalence reduction has not occurred. The increase in the use of statins has led to significant reduction in population cholesterol levels [23]. Likewise, more and better treatment of hypertension has led to less hypertension and associated illness such as heart attacks and strokes illness [24, 25]. At least seven hypotheses can explain why more and better treatments have not reduced common mental disorder prevalence:

1. Increased willingness of individuals to report symptoms and pressures to diagnose distress as anxiety or depression has inflated prevalence rates and masked a true treatment-driven prevalence drop (further: diagnostic inflation).

2. Mood and anxiety disorders first incidence has increased and offset a treatment-driven prevalence drop.

3. Randomized controlled trials (RCTs) have overestimated the acute-phase treatment efficacy and so have [4] treatments targeted at maintaining acute-phase treatment gains.

5. Trial efficacy does not generalize to real-world effectiveness.

6. Treatment has benefited nonrecurrent/nonchronic cases more than chronic-recurrent cases while treatment's population-level impact is much larger for the latter.

7. Counterproductive effects of treatment have reduced its effectiveness to impact at the population level.

## Diagnostic Inflation

Willingness of individuals to present distress in treatment settings on the one hand and over-medicalization by providers on the other may have increased in recent decades [22, 26, 27, 28]. In combination with the lack of physiological criteria, the imperfections in measurement and diagnostic systems including the increase of diagnostic entities and changes in diagnostic criteria [29, 30, 31], these trends could have inflated prevalence rates in epidemiological studies [32]. Stable prevalence rates would then mask a true treatment-driven prevalence drop.

Although it is likely that the trends of increased willingness and medicalization have inflated prevalence rates in general medical settings [22, 28, 33], the evidence suggests that it is less likely that any systematic drift in “caseness” has occurred in population-based epidemiologic surveys if these surveys have been conducted by well-trained interviewers using structured interviews (e.g., Diagnostic Interview Schedule, DIS; Composite International Diagnostic Interview, CIDI) to generate well-standardized diagnostic classifications (e.g., DSM-3 and −4) [29, 30, 31, 34, 35]. Thus, it is unlikely that an increase in false positives has masked a treatment-driven drop in “true” prevalence.

## First Incidence Has Increased and Offset Treatment-Driven Prevalence Drop

Another obvious explanation is that a rise in first incidence has offset the expected treatment-driven decrease in prevalence of mood and anxiety disorders. First incidence refers to the percentage of individuals in the general population that meet the diagnostic criteria for a particular disorder for the first time in their life during a particular period, usually a year (annual first-incidence rate); lifetime prevalence refers to the percentage of individuals in the general population that meet diagnostic criteria for a particular disorder at least once during their lifetime and thus equals first incidence during lifetime. Table 1 presents incidence rates of mood and anxiety disorders as observed in the few post-1980 first-incidence studies of epidemiological samples that meet the following criteria: *N* ≥ 1,000, operationally defined diagnostic classification, standardized psychiatric interview administered by well-trained interviewers, and follow-up periods of ≤3 years. If the first incidence has increased since the 1980s, the annual rates should show a consistently increasing trend over the years. The annual incidence rates from the periods 1981–1982, 1997–1999, 2004–2006, and 2008–2011 do not suggest a consistent increase. The incidence rates of major depression, generalized anxiety disorder, and panic disorder show a temporary increase in the late 1990s whereas the incidence rate of social anxiety disorder has decreased. However, it should be stressed that the evidence on first incidence is scarce, heterogeneous, and ends around the early 2010s [36]. We conclude that it is unlikely that a significant rise in first incidence has offset a treatment-driven prevalence drop, but the data are too limited for any definite conclusion [36].

## Acute-Phase Treatment Efficacy

Leading clinical practice guidelines on anxiety and depression indicate that antidepressants and benzodiazepines and/or any of several empirically supported psychological treatments are efficacious. However, if treatment efficacies are more modest than guidelines suggest, it is possible that, even with more people receiving gold standard interventions, any decrease in prevalence would be small and elusive. This could help to explain the treatment-prevalence paradox.

Although meta-analyses and umbrella reviews have serious limitations for clinical practice [37, 38, 39], they are useful to illustrate the impact of methodological weaknesses on efficacy estimates (see Table 2 for important biases). Meta-analyses that adjust for biases show that efficacy is substantially smaller than conventionally believed. Two comprehensive umbrella reviews, published in 2014 and 2022, clearly demonstrate the impact of biases on reported efficacy. The 2014 review [40] reported a medium overall effect size (SMD = 0.50), across psychotherapies and medications. In contrast, the 2022 review [41] found a small overall effect size of 0.34 for psychotherapies and 0.36 for medications. The 2022 review included only RCTs that had used placebo or care-as-usual as comparison group, formally assessed study quality, and included meta-analyses published since 2014. Individual meta-analyses that adjust for risk of bias report substantial drops in efficacy [42, 43].

What do such modest efficacies mean for the treatment-prevalence paradox? Here, we must realize, first, that what matters from the population perspective is efficacy relative to no treatment and not relative to placebo or care-as-usual. Both control conditions likely have slightly better outcomes than no treatment but how much better is unknown because no-treatment control groups are rare [44]. The second caveat is that adjustment has typically been limited to publication bias and exclusion of trials that do not report intention-to-treat analysis or include wait list control groups. Efficacies could even be smaller as the cumulative effect of all biases is unclear [45]. Especially, unblinding remains difficult to quantify validity threat, obviously for psychotherapy trials, but for medication trials as well. Hence, it is possible that even the efficacy relative to no treatment is too small to matter at the population level and if true helps to explain the treatment-prevalence paradox.

## Efficacy of Interventions to Prevent Relapse and Recurrence

Many patients do not maintain their acute-phase treatment response, about a fifth (psychotherapy) to a third (medication) relapse within 1 year [46, 47, 48, 49, 50]. To prevent relapse recurrence, interventions were developed and indeed substantial benefits have been reported for continued medication and preventive psychotherapy relative to controls although the evidence for anxiety disorders is limited. Continuing antidepressant medication halves risk of relapse/recurrence relative to substitution to PLA within the first year [48, 49, 51, 52]. Psychotherapies are significantly better than routine clinical management in reducing relapse/recurrence risk in patients who are at least in partial remission at randomization; interestingly, they seem also slightly more successful than continued medication [53]. Most follow-ups lasted 12–24 months.

However, methodological concerns remain, complicating interpretation, including misclassification of medication withdrawal symptoms, unblinding, heterogeneity of control conditions, and therapeutic allegiance problems. In addition, two other issues are relevant as well. First, patients without response to acute-phase treatment were not eligible for these trials. Second risk of relapse/recurrence, albeit substantially reduced by continued medication or preventive psychotherapy, remains significant [48, 54]. Despite these interpretation problems, if widely and adequately implemented in real-world settings, relapse-recurrence preventive interventions should have some impact at the population level.

## Does RCT's Efficacy Generalize to Routine Care?

The limited RCT-based treatment efficacy may not generalize well to “real-world” practice: the well-recognized distinction between treatment *efficacy* as established in RCTs (i.e., under optimal conditions) versus treatment *effectiveness* as realized in routine care (i.e., under typical conditions) [55]. First, the typical patient in routine care may have two additional disadvantages compared to his RCT counterpart: poorer prognosis and less optimal treatment [49]. Indeed, remission rates in routine practice are substantially lower than in meta-analyses for all treatment modalities (32% vs. 40–74%) [56].

In addition, research indicates substantial treatment gaps in the dissemination and implementation of treatment protocols [57, 58, 59, 60, 61, 62, 63, 64]. The WHO World Mental Health surveys reported that the 12-month service use among the 1,238 participants from 10 high-income countries with a *severe* 12-month CIDI-DSM-IV anxiety, depressive, or substance use disorder ranged from 24% to 61% (mean 55%). Only on average 35% of these service users had received minimally adequate treatment [65]. Other studies report similar observations [66, 67]. Another problem is that some RCT-based treatments are so highly specialized that they are difficult to implement into routine care. Although the consequences of these gaps are not fully clear [68, 69], the conclusion seems inescapable that not only the efficacy of treatment has been overestimated but also its generalizability to routine care settings. Thus, poor generalizability helps to explain the treatment-prevalence paradox.

## Nonoptimal Targeting of Treatment and the Population Significance of Recurrent-Chronic Cases

Even with better treatments being more widely available, its impact on the prevalence is dependent on how optimal they are targeted. Crucial here is the distinction between recurrent-chronic cases and those with one or two lifetime episodes (nonrecurrent cases) since effective treatment of recurrent-chronic cases has much more impact at the population level than effective treatment of nonrecurrent cases, even if the nonrecurrent cases by far outnumber the recurrent-chronic cases [70, 71, 72, 73, 74]. The reason for the larger population-level impact is that recurrent-chronic cases make up the majority in *prevalence* rates as the total time during their life that they meet diagnostic criteria is much longer than for the nonrecurrent cases because of which they have a much higher probability to be picked up in epidemiological prevalence studies.

The recurrence issue has two, potentially important, implications or scenarios. First, if the RCTs on the efficacy of treatments have largely been obtained on episodes of nonrecurrent-nonchronic cases and not on those of recurrent-chronic cases, efficacy may have been overestimated as by all clinical accounts, effective treatment of recurrent-chronic cases is more difficult [75, 76]. Second, if in routine care relatively efficacious treatments to prevent relapse/recurrence have not been optimally targeted, the impact at the population level will have been small.

It remains unclear to what degree these two scenarios reflect past clinical practice. If frequently and adequately provided, therapeutic advances for preventing relapse/recurrence should have produced a robust treatment-induced prevalence drop. If rarely or nonoptimally provided, it could help explain the treatment-prevalence paradox.

## Can Treatment Be Counterproductive?

Both medication and psychological treatment of mood and anxiety disorders can have adverse effects [77]. How frequently these adverse effects occur and outbalance the benefits of treatment is less clear. In particular, medication has been associated with a variety of adverse effects: paradoxical effects, manifestations of tolerance (loss of clinical effect, refractoriness), withdrawal symptoms and disorders [78, 79, 80, 81]. Significant adverse effects of psychological treatments have been noted as well [82, 83].

Two important counterproductive consequences have been proposed: reduction of self-help activities and loss of agency coping [84] and behavioral toxicity including oppositional perturbation and symptom return [77, 78, 80, 81]. They merit consideration as possible contributors to the treatment-prevalence paradox.

*Reduction of self-help activities and loss of agency*: medication treatment without behavioral management and psychoeducation (mono-medication) has the risk of being counterproductive if it reduces self-help activity and active coping [84]. The same might apply to low-fidelity-to-guideline psychotherapy [80]. The argument is that depressed and anxious people often engage in helpful strategies subsumed in self-help programs and psychological treatments, such as exercising, increasing pleasant activities, reducing stressful situations, and meditating, which tend to improve their “agency,” “self-efficacy” for coping with underlying problems [32], and perhaps even their neural plasticity [85]. Mono-medication and low-fidelity-to-guideline psychotherapy have the risk to reduce these helpful strategies.

*Behavioral “toxicity” including oppositional perturbation*: behavioral toxicity refers to the “pharmacological actions of a drug that, within the dose range in which it has been found to possess clinical utility, may produce alterations in mood, perceptual, cognitive and psychomotor functions, that limit the capacity of the individual or constitute a hazard to his/her well-being” ([77], p.130). An important form of behavioral toxicity is “oppositional perturbation” that seeks to account for unintended and unwanted effects of medication on illness course, including symptom return after discontinuation, and a progressive loss of effectiveness (tachyphylaxis) across repeated anti-depressant medication trials [78, 79, 80, 81]. Importantly, *direct* evidence for oppositional perturbation is lacking, but intriguing *indirect* evidence is available [78, 79].

RCTs have not actively searched for adverse consequences, perhaps because it was not in the interest of the funding bodies or researchers. Medication monotherapy and low-fidelity-to-guideline psychotherapy are probably uncommon in RCTs, where treatment protocols are specified and carefully monitored, unlike treatment in real-world settings. In addition, no-treatment arms are considered unethical and medication-withdrawal studies may have missed the bigger picture of improved ultimate outcomes, due to misinterpreted withdrawal symptoms and too short follow-ups [79, 86]. In conclusion, although hard data are lacking, counterproductive effects could significantly help to explain the treatment-prevalence paradox, but solid evidence is lacking.

## Concluding Comments

Since the early 1980s, mental health care expenditures for mood and anxiety disorders have increased substantially in the Western world, especially treatment with medications, typically serotonin reuptake inhibitors. However, reductions in the prevalence of mood and anxiety disorders have not accompanied this expansion, the treatment-prevalence paradox. Our analysis suggests that it is unlikely that substantial increases of false positives or first incidence have offset a true treatment-driven reduction in prevalence. Instead, it seems likely that the treatment-prevalence paradox is, at least in part, due to overrated efficacy of treatment and major quality gaps in routine care settings. In addition, it is possible that nonoptimal targeting of treatment (too little on recurrent and chronic cases) and counterproductive effects of treatment account for part of the paradox, but convincing evidence is lacking. Compared to short-term outcome, much less is known about long-term outcome and outcome in terms of quality of life and socio-economic functioning. Thus, more research on long-term outcome, optimal treatment targeting, and counterproductive treatment effects is crucial. The overoptimistic view of treatment efficacy in clinical guidelines is not only due to significant methodological weakness in RCTs and meta-analyses but to publication, outcome reporting, spin, and citing bias as well [45]. In addition, co-morbidity has hardly been addressed in treatment studies. There is a clear need of treatment studies taking into account the co-morbidity of the patients involved, not only of co-morbid anxiety and mood disorders but of substance abuse as well [87].

To reduce mood and anxiety disorders prevalence not only more efficacious, better implemented and targeted treatments are needed but also *prevention* given the limited population-level impact of treatment and the substantial continuity of psychopathology across childhood, adolescence, and adulthood [88, 89, 90, 91]. If prevention could interrupt this continuity and turn around maladaptive pathways, prevalence would drop substantially. In order to be successful, various authors have argued that effective prevention will have to be structural, well-funded, long-term, socially embedded, starting at an early age, addressing both parenting, kids, and schools, and combining universal (health promotion) and indicated/selective prevention [15, 92, 93, 94]. In short, we need a paradigm shift in both treatment, prevention, and their evaluation to reduce mental disorder prevalences [15, 93].

## Conflict of Interest Statement

All authors report no financial interests or potential conflicts of interest.

## Funding Sources

This paper was supported by a subgrant from the Netherlands Organization for Scientific Research to the first author (Mother Grant NWO-Gravitation 024.001.003).

## Author Contributions

Ormel conceived and wrote the first draft of the manuscript and the revision. Emmelkamp added relevant data on anxiety disorders and improved the revision.

## Figures and Tables

**Table 1:** Annual incidence per 1,000 pyar of post-1980 incidence studies of CMDs meeting the inclusion criteria^a^

	Country, study name, and reference			
	USA, ECA [Eaton, 1989, 1994]	NL, NEMESIS-1 [Bijl, 2002]	USA, NESARC [Grant, 2009]	NL, NEMESIS-2 [de Graaf, 2013]
Sample size/pyar	~10,861 (18+)	4,757 (18–64)	28,614 (18+)	~12,311 (18–64)
Data collection follow-up	1981–1982	1997–1999	2004–2006	2008–2011
	Annual incidence per 1,000 pyar (s.e.; 95% CI)		
MDD	15.9 (1.7)	27.2 (22.6–31.9)^b^	15.2 (0.9)^b^	15.8 (13.6–18.0)^b^
GAD	−	7.3 (5.0–9.4)	11.3 (0.8)	6.4 (5.1–7.7)
PAN	5.6 (0.9)	7.8 (5.5–10.1)	6.2 (0.5)	5.3 (4.1–6.4)
SOC	9.4 (7.4–11.4)	9.3 (6.7–11.9)	3.2 (0.4)	4.1 (3.0–5.1)
Any anxiety disorder	−	−	15.8 (0.9)	16.9 (14.6–19.2)

a Inclusion criteria: post-1980 prospective follow-up study of community-based sample, sample size 1,000+, operationally defined diagnostic classifications (e.g., DSM-3 or 4), standardized psychiatric interview administered by experts or trained lay interviewers, follow-up up to 3 years. pyar, person-years-at-risk; MDD, major depressive disorder; GAD, generalized anxiety disorder; PAN, panic disorder; SOC, social anxiety disorder.

b Difference in MDD incidence between NEMESIS-1 (DSM-3-R) and the other two studies (NEMESIS-2 and NESARC, both DSM-4) might be due to difference between these DSM editions.

**Table 2:** Bias in RCTs and their meta-analyses

Type of bias	Description
*Selection bias* (*inappropriate random sequence generation and/or inappropriate concealment of allocation*)	Biased allocation to interventions due to inadequate generation of a randomized sequence and/or biased allocation to interventions due to inadequate concealment of allocations before assignment

*Selective reporting* or *outcome reporting bias*	Failure to describe negative findings within a published report or switching the status of (nonsignificant) primary and (significant) secondary outcomes

*Outcome misclassification bias*	Measures and assessors are imperfect. In studies that discontinue ADM, withdrawal symptoms may masquerade as depressive symptoms, thereby conflating the two

*Imperfect blinding*	Patients, treatment providers, or assessors know the true status of the randomized subjects: intervention or control condition. In ADM trials, this may occur because of side effects

*Spin bias*	Reporting strategies in a manner that often misleads readers

*Citation bias*	Trials with positive results receive more citations than negative studies, leading to a heightened visibility of positive findings and reduced discoverability of negative trials

*Completer analysis* bias	Only individuals who completed the treatment and post-treatment assessment are included in the analysis because treatment completion is not random and probably dependent on the nature of treatment and control condition; results can be biased, typically in favor of the experimental treatment. These risks are avoided when using intention to treat, i.e., using all persons randomized

*Inappropriate controls*	Controls do not fully meet the objectives of the study. For instance, if no treatment is the best control condition given the study objectives then wait-list (nocebo effect) and treatment as usual (heterogeneous) are imperfect controls
